# Impaired Axonal Na^+^ Current by Hindlimb Unloading: Implication for Disuse Neuromuscular Atrophy

**DOI:** 10.3389/fphys.2016.00036

**Published:** 2016-02-16

**Authors:** Chimeglkham Banzrai, Hiroyuki Nodera, Toshitaka Kawarai, Saki Higashi, Ryo Okada, Atsuko Mori, Yoshimitsu Shimatani, Yusuke Osaki, Ryuji Kaji

**Affiliations:** Department of Neurology, Tokushima UniversityTokushima, Japan

**Keywords:** axonal excitability, disuse atrophy, persistent sodium current, ion channels, threshold tracking

## Abstract

This study aimed to characterize the excitability changes in peripheral motor axons caused by hindlimb unloading (HLU), which is a model of disuse neuromuscular atrophy. HLU was performed in normal 8-week-old male mice by fixing the proximal tail by a clip connected to the top of the animal's cage for 3 weeks. Axonal excitability studies were performed by stimulating the sciatic nerve at the ankle and recording the compound muscle action potential (CMAP) from the foot. The amplitudes of the motor responses of the unloading group were 51% of the control amplitudes [2.2 ± 1.3 mV (HLU) vs. 4.3 ± 1.2 mV (Control), *P* = 0.03]. Multiple axonal excitability analysis showed that the unloading group had a smaller strength-duration time constant (SDTC) and late subexcitability (recovery cycle) than the controls [0.075 ± 0.01 (HLU) vs. 0.12 ± 0.01 (Control), *P* < 0.01; 5.4 ± 1.0 (HLU) vs. 10.0 ± 1.3 % (Control), *P* = 0.01, respectively]. Three weeks after releasing from HLU, the SDTC became comparable to the control range. Using a modeling study, the observed differences in the waveforms could be explained by reduced persistent Na^+^ currents along with parameters related to current leakage. Quantification of RNA of a SCA1A gene coding a voltage-gated Na^+^ channel tended to be decreased in the sciatic nerve in HLU. The present study suggested that axonal ion currents are altered *in vivo* by HLU. It is still undetermined whether the dysfunctional axonal ion currents have any pathogenicity on neuromuscular atrophy or are the results of neural plasticity by atrophy.

## Introduction

Skeletal muscle atrophy because of inactivity and immobilization (disuse muscle atrophy) poses substantial problems to the affected individual and society. The increased need for support and the risk for falls and disability lead to increasing healthcare costs in aging societies. Despite the imminent need for proper prevention and treatment of disuse muscle atrophy and weakness, its pathophysiology has only been partially elucidated. More exactly, pathophysiology for abnormal regulation of skeletal muscle size has been elucidated in detail, including a reduction in muscle protein synthesis and an increase in protein degradation. Importance of oxidative stress and dysfunction of muscle mitochondria for etiology in disuse muscle atrophy has been reported (Wiggs, 2015). On the other hand, attention has been paid less to potential roles of innervating motor axons for the pathogenesis of disuse muscle atrophy. One of the animal models for disuse atrophy, denervation model, has shown dysfunctional mitochondrial protein import that implies significant roles of neurotrophic support to maintain a muscle size (Singh and Hood, 2011).

Diffuse muscular atrophy might be present in patients with critical illness and can be due to either dysfunction of muscle itself (critical illness myopathy) or motor axons (critical illness neuropathy) or both. The pathomechanisms for these have been elucidated to be multifactorial, but include inflammation-induced catabolic state, sepsis, multiorgan failure, and bed rest (Poulsen, 2012; Hermans and Van den Berghe, 2015), thus sharing some pathophysiologies in common with disuse muscle atrophy.

Proper regulation of neuronal excitability is a key to maintain neuronal environment. In amyotrophic lateral sclerosis (ALS), a neurodegenerative disease affecting upper and lower motor neurons, hyperexcitability of motor neurons and axons have been reported (Kanai et al., 2006). Dysfunctions of neuronal ion channels play a major role in its hyperexcitability, especially the ones that are open at the subthreshold levels. Upregulation of persistent Na^+^ current and downregulation of “slow” K^+^ current have been reported in ALS (Kanai et al., 2006; ElBasiouny et al., 2010). Consequently, hyperexcitability in various diseases such as ALS and sensory neuropathy by mutated sodium channels causes impairment of energy deficit, abnormal influx of Ca^2+^, dysfunctional neuronal mitochondria, and neural death (Estacion et al., 2015; Ngo and Steyn, 2015).

On the other hand, disuse and immobilization of a limb cause plastic changes of diffuse areas in the central and peripheral nervous systems. Hypoexcitability by disuse and immobilization has been reported by neurophysiological studies and neuroimaging techniques (Taniguchi et al., 2008; Langlet et al., 2012; Rosenkranz et al., 2014). Several lines of evidences imply potential neurodegenerative effects by hypoexcitability, such as impaired axonal transport and proper localization of mitochondria resulting in energy failure (Andrews et al., 2005; Chen and Sheng, 2013).

Hindlimb unloading (HLU) in animals has been used as a model for disuse neuromuscular atrophy. Previous studies have suggested dysfunction at the level of muscle fibers, motor axons, and motoneurons. Neurophysiologically, the observed abnormal motor unit firing rates, contractile properties, and physiological properties suggest impaired neuroaxonal excitability (Duchateau and Hainaut, 1990; Cormery et al., 2005). However, the pathogenesis of the neuronal dysfunction caused by HLU remains to be elucidated. Given the significance of regulating neuronal excitability which is highly activity-dependent and its potential association with neuromuscular cell death, we hypothesized that dysfunctions of axonal ion channels are present in the motor system by disuse, such as impairment of axonal Na^+^ and K^+^ currents. Threshold tracking is a non-invasive neurophysiological test similar to nerve conduction study in humans which applies various durations and polarities of conditioning electrical pulses and “track” the resulting excitability changes of the peripheral motor (or sensory) axons (Bostock et al., 1998). Computer modeling can quantify membrane properties such as the resting membrane potential (RMP) and axonal ion currents (Kiernan et al., 2005; Shimatani et al., 2015).

Thus, the aim of the present study was to test the hypothesis that axonal ion currents are rendered dysfunctional by HLU.

## Methods

### Hindlimb unloading

The experiment was approved by the institutional Animal Care and Use Committee at Tokushima University and was carried out in accordance with the Council Directive 2010/63EU of the European Parliament and the Council of 22 September 2010 on the protection of animals used for scientific purposes. All studies were conducted in accordance with the United States Public Health Service's Policy on Humane Care and Use of Laboratory Animals. ICR male mice (SLC, Hamamatsu, Japan), 8-weeks-old, were tested. The tail of each mouse was cleaned, dried, and wrapped in adhesive tape. Then the animal was unloaded by tail suspension using a tail clip (Yamashita Giken, Tokushima, Japan). The body was maintained at a 30° angle with the head pointed down and care was taken to ensure that the mice did not contact the ground with their hindlimbs. The animals were free to move their forelimbs and were fed *ad libitum*. Mice in the HLU group were maintained in this manner for 3 weeks prior to conducting the experiments. The adhesive tape was changed every 10 days to prevent ischemia of the tail. Age-matched mice without intervention were used as controls.

### Axonal excitability study

After inducing anesthesia with isoflurane at 1.5% for 30 min, electrophysiological studies were performed. The animal was warmed on a thermostat-controlled heating pad (BWT-100A, Bioresearch Center, Nagoya, Japan) to maintain the hindlimb temperature at 33–34°C throughout the studies. Compound muscle action potentials (CMAPs) of the sciatic nerve were recorded by placing 30-gage stainless steel, disposable needle electrodes in the plantar muscle (for active recording), and dorsum of the ipsilateral foot (for reference). The cathode and anode of the wire surface electrodes were placed above the ankle of the hindlimb and at the base of the tail. The ground needle electrode was placed midway between the stimulating and recording electrodes (Boërio et al., 2009).

For neuronal excitability testing, stimulation was controlled by a PC running the QtracS program (Institute of Neurology, London), connected via a digital I/O device (National Instruments, Austin, TX) to a preamplifier (MEG-1200: Nihon Kohden, Tokyo, Japan) and a stimulator (DS-4: Digitimer, Letchworth, UK). Using 1-ms rectangular stimuli, the negative peak of the CMAP was recorded. For excitability tests, the TRONDNF multiple excitability recording protocol was used. Stimulus-response curves, which were determined using a 1-ms duration test stimulus, increased from zero until supramaximal potentials were attained. To record threshold electrotonus (TE), the unconditioned threshold at one channel was tracked, while that at discrete points was determined at three other channels as follows: (1) during and after 100-ms of hyperpolarizing and depolarizing currents, the conditioning currents were set to ±40% of the unconditioned threshold, and (2) during and after 200 ms of hyperpolarizing current, set to −70% of the unconditioned threshold. For the +40% depolarizing conditioning current, the difference of threshold changes between the greatest threshold reduction and at the end of the 100-ms conditioning pulse was defined as S2 accommodation. For the −70% hyperpolarizing conditioning current, the lowest threshold reduction was defined as the TEh (peak: −70%). The difference of threshold changes between the TEh (peak: −70%) and at the end of the 200-ms conditioning pulse was defined as S3 accommodation. For the recovery cycle (RC), a supramaximal conditioning stimulus was given with delays ranging from 200 to 1.6 ms before the test stimulus provided at another channel. The current-threshold relationship (I/V) was then recorded with a 1-ms test stimulus applied 200 ms after the onset of a long-lasting subthreshold polarizing current, the strength of which was altered in steps of 10%, from +50% (depolarizing current) to −100% (hyperpolarizing current) of the control threshold. The strength-duration time constant (SDTC) describes the stimulus strength required to excite nerves as stimulus width is increased from 0.2 to 1.0-ms duration. A set of excitability parameters was derived from the recordings as previously described (Nodera and Rutkove, 2012a; Supplemental Figure 1).

### Data analysis for excitability study

Axonal excitability data between the two groups were compared by the Mann-Whitney U test (QtracP, Degitimer, UK). Statistical significance was set at *P* < 0.05.

For modeling of the excitability data, the Bostock model of the human motor axon was used in the simulation of axonal excitability (MEMFit, QtracP version 1/3/2015), as previously explained (Kiernan et al., 2005; Howells et al., 2012). Parameter adjustments were made to improve the fit to RC, SDTC, I/V, and TE with the same weighting on these tests. The tested parameters were as follows: nodal and internodal resting potentials, nodal sodium permeability, percent persistent Na^+^, nodal and internodal slow K^+^ conductance, nodal and internodal fast K^+^ conductance, internodal H conductance, nodal and internodal leak conductance, Barrett-Barrett conductance, and total pump currents.

### Quantification of RNA

After the axonal excitability studies were terminated, mice were terminally anesthetized by CO_2_ and the bilateral sciatic nerves were excised.

Total RNA was extracted from excised sciatic nerves from hinder-limb-hanged and control mice (*N* = 6, respectively). Real-time quantitative reverse transcription–PCR (RT-PCR) of α-subunits of axonal sodium channels (SCN1A for Na_*v*_1.1 and SCN1B for Na_*v*_1.2) was performed using specific primers. Total RNA was extracted from excised sciatic nerves of more than 1 cm from HLU and control mice, using RNAiso Plus reagent (TaKaRa Bio, Inc. Kyoto, Japan) according to the manufacturer's instructions. Quantity and quality of RNA were evaluated using NanoDrop 1000 (Thermo Fisher Scientific Inc., MA, USA). cDNA was prepared from 5 mg of total RNA, with random hexamer primers and the PrimeScript II 1st strand cDNA synthesis Kit (TaKaRa Bio, Inc. Kyoto, Japan). Real-time quantitative reverse transcription–PCR was performed using the SYBR Premix Ex Taq II (Takara Bio, Japan) and specific primers (Supplemental Table 1). The qRT-PCR products were validated using agarose gel electrophoresis and Sanger sequencing as described elsewhere (Kawarai et al., 2015). The efficiency of qRT-PCR was evaluated using melting curve analysis using the defaulted program of the StepOne Plus. One housekeeping gene, mouse ribosomal protein S16 was selected as internal control genes to normalize the PCR. Data of relative expression level were analyzed with the 2^−Δ*ΔCP*^ method (Livak and Schmittgen, 2001). Quantitative PCR (qPCR) was carried out in a real-time PCR system (StepOnePlus, Life Technologies) and Relative Quantitation (RQ) values were simultaneously evaluated using the installed software (StepOneSoftware, version 2.3; Life Technologies). Statistical analysis was performed using SPSS 22 (SPSS Inc., USA) software. The Mann-Whitney U test was performed to compare the values between the groups, given the relatively small number of the subjects. The limit for statistical significance was set at *P* = 0.05.

## Results

### Characteristics of the animals

Despite the similar baseline weights between the groups [31.0 ± 0.9 g (HLU) vs. 30.6 ± 0.7 g (Control), *P* = 0.2], the HLU group weighed lesser than the age-matched controls [34.2 ± 1.7 g (HLU) vs. 44.1 ± 3.8 g (Control), *P* < 0.0001], as reported previously (Ohno et al., 2014). The amplitudes of CMAPs were significantly lower in the HLU group than in the control [2.2 ± 1.3 mV (HLU) vs. 4.3 ± 1.2 mV (Control), *P* = 0.03]. There was no significant difference in the peak motor latency or the strength of the stimulus current.

### Axonal excitability study

**Figure 1:**
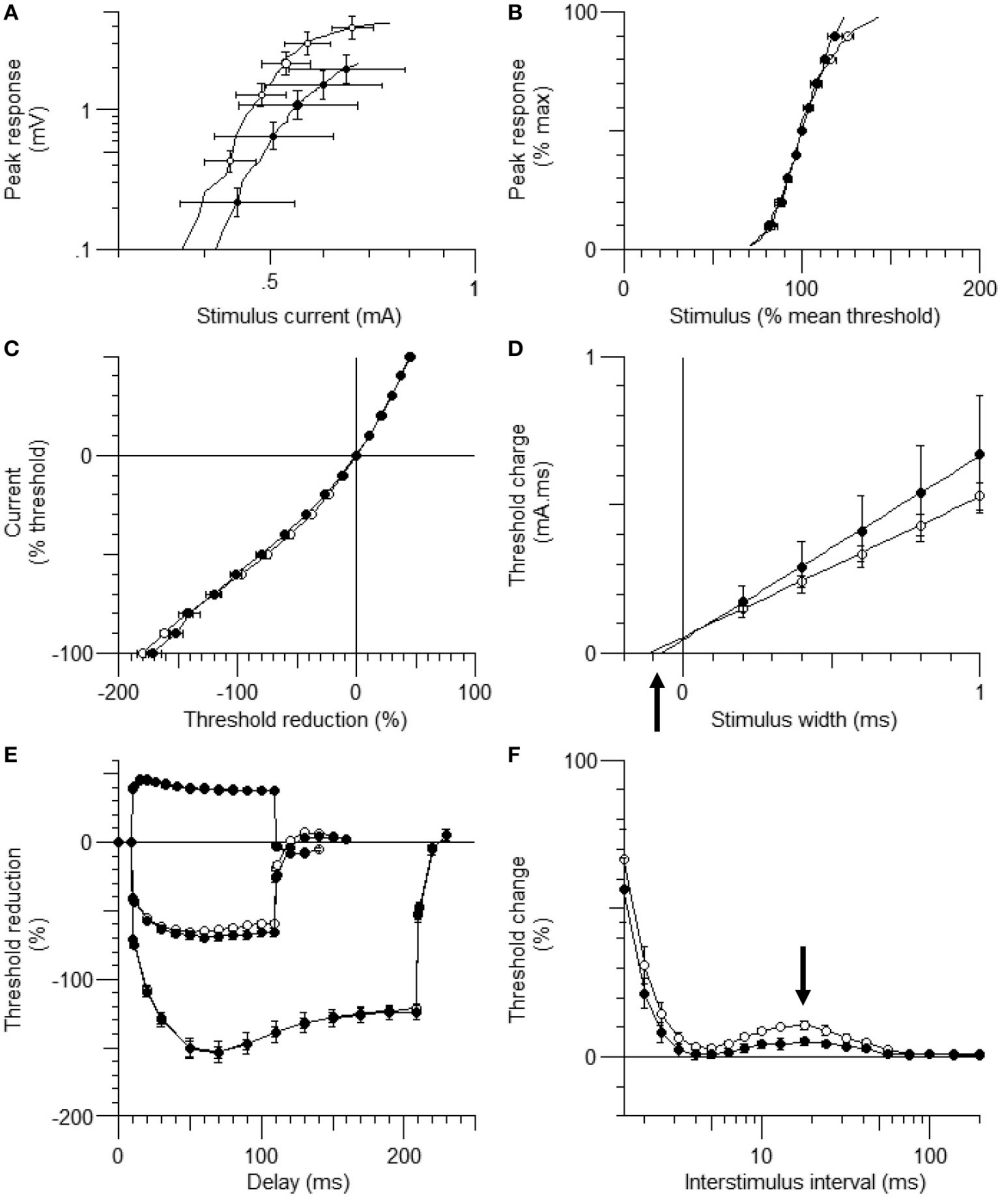
**The waveforms of axonal excitability studies**. The data are shown as mean ± standard error of the mean [filled circles = hindlimb unloading (HLU) (*N* = 14): open circles = control (*N* = 10)]. The mean amplitude of the compound muscle action potential (CMAP) in the HLU group was approximately half of the control group (*P* = 0.03) and tended to require stronger currents **(A)**. The relative slopes of the current-response curves were similar **(B)**. The waveforms of threshold electrotonus and current/threshold relationship (I/V) were similar **(C,E)**. Strength-duration time constant (SDTC) was smaller in the HLU group than in the control (*P* < 0.01) (**D**, the intercept with the x-axis, arrow). Recovery cycle showed smaller late subexcitability in the HLU group (**F**, arrow).

Figure 1 shows the waveforms of the axonal excitability study. The data obtained from the control group were similar to those in a previous study (Table 1; Boërio et al., 2009). The threshold changes by long depolarizing and hyperpolarizing currents were similar between the two groups [Figures 1C,D, current-threshold relationship (I/V) and threshold electrotonus], although the S2 accommodation tended to be smaller in the HLU group than in the controls. RC showed similar refractoriness, relative refractory period, and superexcitability. The late subexcitability and SDTC were smaller in the HLU group than in the controls (*P* = 0.01 and < 0.01). To identify the interval excitability changes after releasing from HLU, an additional experiment was carried out in the separate groups of animals (*N* = six each, 3 weeks of HLU and control). The lower SDTC immediately after HLU for 3 weeks was not present 3 weeks after releasing from HLU, suggesting reversible changes (Supplemental Figure 2).

**Table 1:** **The parameters of the motor conduction and the excitability study**.

	**Unloading (*N* = 14)**	**Control (*N* = 10)**	***P* values**
CMAP amplitude (mV)	2.2 ± 1.3	4.3 ± 1.2	0.03
Peak latency (ms)	1.9 ± 0.1	2.1 ± 0.1	0.12
Stimulus for 50% maximum amplitude	0.55 ± 1.2	0.52 ± 1.1	0.8
**THRESHOLD ELECTROTONUS**			
TEd (10–20 ms)	44.0 ± 1.8	44.5 ± 0.8	1.0
TEd (40–60 ms)	39.0 ± 1.2	38.8 ± 0.5	0.7
TEd (90–100 ms)	37.8 ± 1.4	37.4 ± 0.7	0.4
TEh (10-20 ms)	−60.3 ± 2.0	−58.7 ± 1.0	0.5
TEh (20–40 ms)	−66.1 ± 2.6	−63.9 ± 1.4	0.4
TEh (90–100 ms)	−65.9 ± 3.0	−59.5 ± 1.7	0.1
S2 accommodation	6.5 ± 1.2	7.2 ± 0.8	0.4
TEh (peak: −70%)	−153.9 ± 8.0	−154.8 ± 5.0	0.9
S3 accommodation (−70%)	29.9 ± 4.5	32.5 ± 2.4	1.0
**RECOVERY CYCLE**			
Relative refractory period	3.3 ± 1.1	2.7 ± 1.0	0.4
Refractoriness at 2 ms	21.3 ± 5.1	31.1 ± 5.3	0.2
Superexcitability (%)	−1.9 ± 0.7	−0.7 ± 0.3	0.2
Late subexcitability (%)	5.4 ± 1.0	10.0 ± 1.3	0.01
**CURRENT/THRESHOLD RELATIONSHIP**			
Resting I/V slope	0.85 ± 0.02	0.91 ± 0.02	0.15
Minimum I/V slope	0.46 ± 0.04	0.44 ± 0.01	0.6
Hyperpolarizing I/V slope	0.53 ± 0.04	0.54 ± 0.02	0.9
Strength-duration time constant	0.075 ± 0.01	0.12 ± 0.01	< 0.01

CMAP, compound muscle action potential. Significant values are underlined.

### Computer modeling

**Figure 2:**
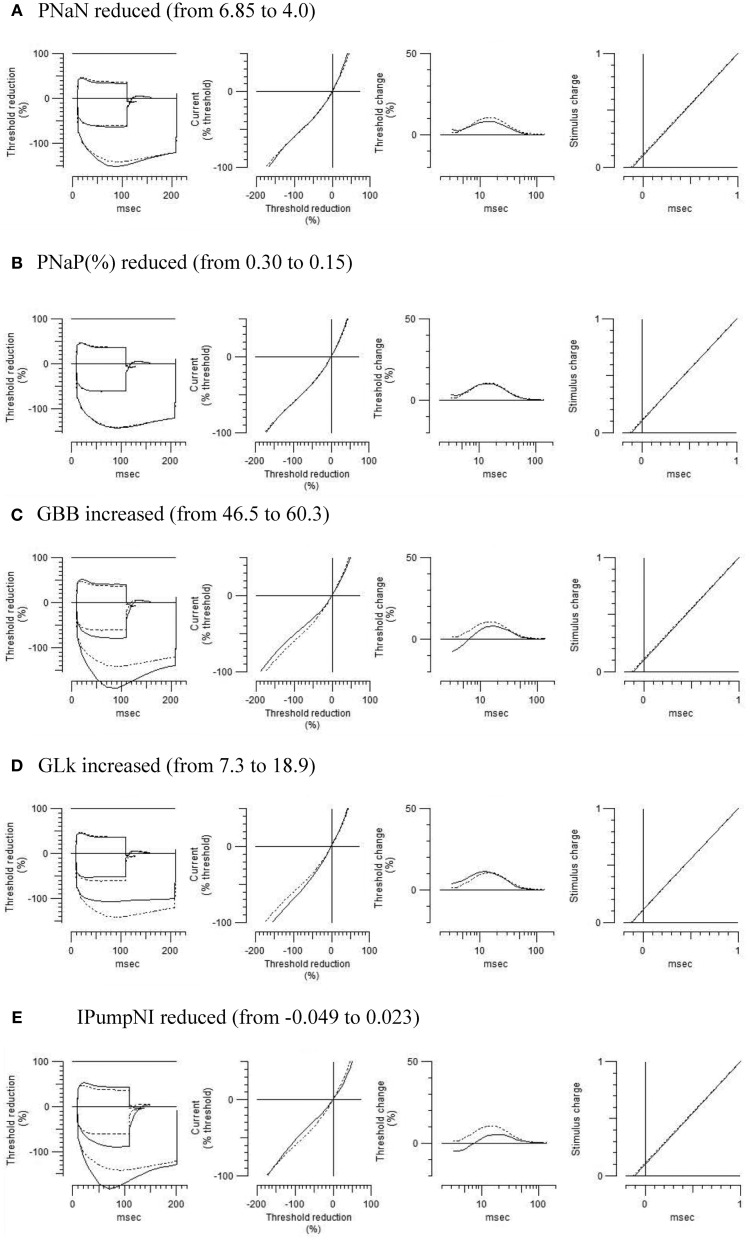
**Modeled effects on multiple excitability tests by changing one of the relevant excitability parameters listed in Table 2 (original control waveforms in dashed line)**. Waveforms in solid line were based on the optimized excitability parameters for the HLU animals as shown in Table 2.

**Figure 3:**
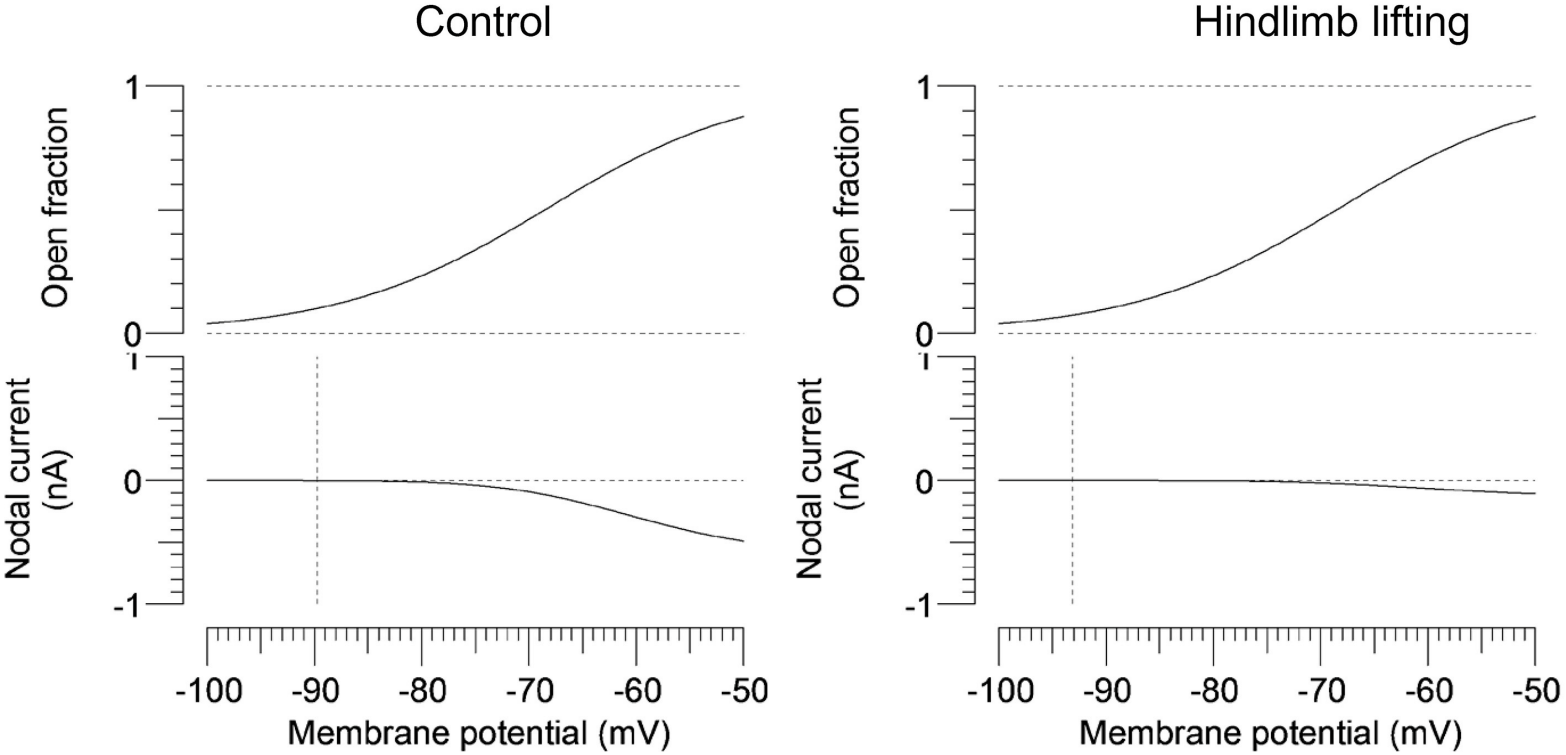
**Modeled gating property and currents of persistent Na^+^ currents**. Voltage-dependent open fractions of m2^3^ gates are comparable between the control (the left panel) and the HLU group (the right panel), whereas the nodal currents are significantly smaller in the HLU group than the controls. Vertical lines indicate modeled resting potentials.

To further characterize the abnormal axonal excitability by HLU, computational modeling was performed to fit the excitability parameters (Supplemental Figure 3). The results of the fitted parameters used to reduce the mean error of the axonal excitability studies were outlined in Supplemental Table 2. Given the complex changes in the parameters, analyses of most responsible parametric changes were further calculated. Changing one parameter from the control values did not satisfactorily fit the waveforms in the HLU group [up to 22.5% error reduction by lowering the percent persistent Na^+^ current, followed by nodal Na^+^ permeability (19.7% reduction) and increasing Barrett-Barrett conductance (GBB) (5.4% reduction)]; therefore, we further modeled the best two and three parameters to explain the difference in the waveforms. The three combinations that maximally reduced the discrepancy are shown in Table 2. Altering two parameters was not satisfactory, because the maximum reduction of error was only 33.4% (Top panel, Table 2). Alteration of three parameters finally yielded the reduction of 80.3% with the combination of the following: (1) decreasing the percent persistent Na^+^ by 87%, (2) increasing the nodal leak conductance by four-fold, and (3) increasing GBB by 39%. The effects of changing each of these parameters on multiple excitability waveforms are shown (Figure 2). Figure 3 compares the kinetics of persistent Na^+^ current, showing similar voltage-dependent open channel fractions between the controls and HLU, but smaller current in HLU, indicating downregulation of INaP without voltage-dependent kinetic changes by HLU.

Table 2**The combinations of two (top) or three (bottom) excitability parameters to find best changes in simulation errors**.

**Table:** 

**Parameter A**	**Final value (original value)**	**Parameter B**	**Final value (original value)**	**% reduction discrepancy**
PNaP (%)	0.014 (0.30)	GBB	52.5 (46.5)	33.4%
PNaP (%)	0.005 (0.30)	IPumpNI	−0.019 (−0.049)	30.9%
GKsI	1000 (272)	GBB	80.4 (46.5)	18.6%

**Table:** 

**Parameter A**	**Final value (original value)**	**Parameter B**	**Final value (original value)**	**Parameter C**	**Final value (original value)**	**% reduction discrepancy**
PNaP (%)	0.04 (0.30)	GLkN	13.9 (1.8)	GBB	64.5 (46.5)	80.3%
PNaN	3.8 (6.85)	GLk	30.5 (7.3)	GBB	76.9 (46.5)	53.8%
PNaP(%)	0.04 (0.3)	GH	55.5 (33)	GBB	58.6 (46.5)	31.8%

*GBB, Barrett-Barrett conductance; GH, Internodal H conductance; GKsI, Max. internodal conductance of slow K^+^ channels; GLk, Nodal and internodal leak conductance; GLkN, Nodal leak conductance; IPumpNI, Nodal and internodal pump current; PNaN, Nodal Na^+^ permeability; PNaP(%), Percent persistent Na^+^*.

### Quantification of RNA

**Figure 4:**
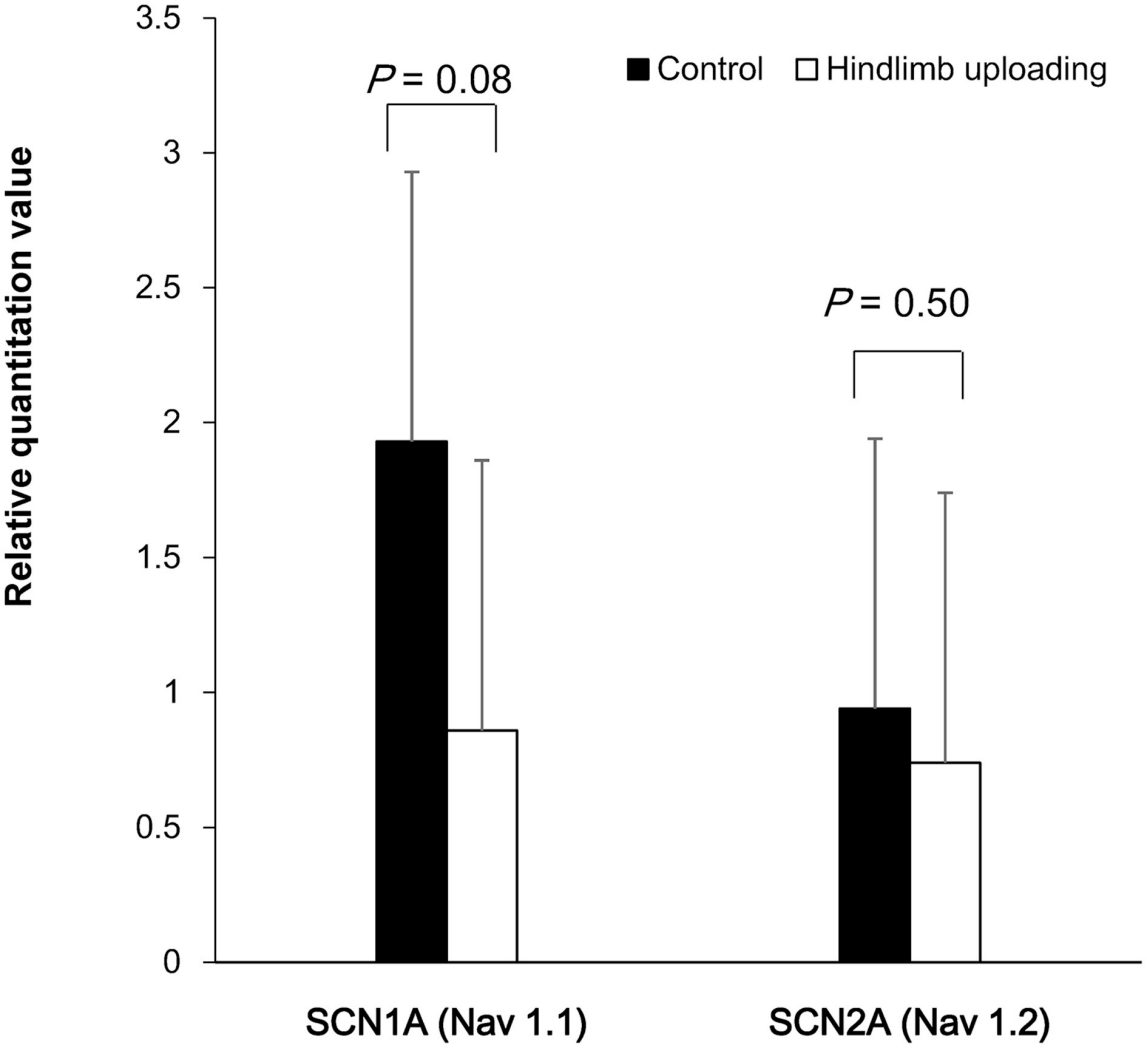
**Quantification of RNA of the SCA1A and SCN2A showed a tendency of lower expression of SCA1A in the HLU group than in the control subjects, whereas the expression of SCA2A were similar between the groups**.

Total RNA was dissolved in 20 microliter of diethyl pyrocarbonate (DEPC)-treated water. The ratio of the absorbance at 260 and 280 nm (A260/280) between 1.85 and 2.06 was obtained for each sample. Agarose gel electrophoresis of the qRT-PCR products showed no detectable non-specific products (Supplemental Figure 4). Melting curve analysis revealed specific DNA fragments. Sanger sequence analysis of qRT-PCR products validated specific amplification of target RNA sequence. Quantification of RNA of SCN1A and SCN2A showed a tendency of lower expression level of SCA1A in the HLU group (*P* = 0.08), whereas the expression levels of SCA2A were similar (Figure 4).

## Discussion

In the present study, we used non-invasive axonal excitability measurements and computational modeling and found dysfunctional ion currents in the peripheral motor axons after HLU. Among the multiple dysfunctional ion currents, reduced persistent Na^+^ currents along with parameters related to current leakage were identified as the candidate parameters, and could explain abnormal axonal excitability by HLU. The dysfunction of Na^+^ currents could be related to the tendency of decreased RNA expression of SCA1A in the HLU group. Additionally, the reduction of axonal “slow” K^+^ current was present, presumably compensatory to maintain proper axonal excitability (see below). It is elusive whether the observed ion channel dysfunctions has any causative roles for neuromuscular degeneration or are just plastic changes to reflect altered neuronal input/out (see below).

### Dysfunction of motoneurons and axons by muscle inactivity

Muscle inactivity by bed rest and space flight causes muscular atrophy (Narici and de Boer, 2011; Sung et al., 2013). Its pathogenesis is multifactorial, but metabolic dysfunctions in the muscle such as impaired protein synthesis and increased proteolysis have been intensely investigated (Phillips and McGlory, 2014; Reid et al., 2014). In addition to causing the primary muscle pathology, inactivity also affects functions of motor neurons and axons. In the peripheral nervous system, reduction of conduction velocities, reduction of maximal firing rate of the motor unit, and altered physiological properties of motoneurons were reported (Duchateau and Hainaut, 1990; Cormery et al., 2005; Clark et al., 2006). In the cerebral cortex, corticospinal excitability was also decreased after bed rest (Roberts et al., 2010). Morphologically, peripheral nerves in the unloaded hindlimb showed reduced myelin thickness (Canu et al., 2009). Overall, these data suggest decreased excitability of the upper and lower motor neurons and their axons by HLU. The possible pathomechanisms are discussed in the following sections.

### Functions of the persistent Na^+^ current

Maintenance of neuronal excitability is crucial for adequate functioning of the nervous system. Excitability is influenced by resting excitability and generation of action potentials. Important parameters that affect excitability are passive membrane properties, transmembrane electrolyte concentrations, ion channels, and pumps (Kiernan and Kaji, 2013).

Voltage-gated Na^+^ channels (VGSC) are responsible for the generation and conduction of action potentials in the motor axons. While majority of the channels get activated and quickly inactivate within a few milliseconds, a small percentage of the Na^+^ channels possess a non-inactivating or slowly inactivating component, called the persistent Na^+^ current (INaP). Because INaP is present near the RMP, INaP defines the RMP and neuronal excitability (Kiss, 2008; Nodera and Rutkove, 2012b). Abnormal INaP has been associated with a number of neurological conditions, such as neuropathic pain, epilepsy, motor neuron disease, axonal regeneration, and diabetic neuropathy (Tamura et al., 2006; Nakata et al., 2008; Misawa et al., 2009; Lauxmann et al., 2013). In ALS, upregulation of INaP was associated with shorter survival and was thus considered a strong predictor of poor prognosis and a potential therapeutic target (Kanai et al., 2012). While upregulation of INaP is associated with neuronal hyperexcitability and death, downregulation of INaP is also reported to be pathogenic, as reported in critical illness polyneuropathy, which shares the physiological features of hypoexcitability observed in inactivity-induced neuropathy (Novak et al., 2009). There have been multiple lines of evidence to suggest causative relationship between inactivity and neuromuscular degeneration through multiple factors as follows. (1) As discussed later, regulation of persistent Na^+^ current is activity-dependent, in part due to alternative splicing (Lin et al., 2012). (2) Decreased mitochondrial axonal transport due to its activity-dependency may lead to its dysfunction and axonal degeneration by intra-axonal Ca^2+^ release (Sajic et al., 2013; Villegas et al., 2014). However, it is premature to conclude whether disuse neuromyopathy could be a direct consequence of suppressed axonal Na^+^ currents. Furthermore, neuronal plasticity of peripheral motor axons has not been confirmed, to our knowledge, except for the level of axonal initial segment (Grubb and Burrone, 2010), however, an *in vivo* experiment suggests activity-dependent plasticity in intrinsic excitability occurring similarly in the axon initial segment and peripheral axons (Rossi et al., 2012).

The present simulation study showed ~5-mV hyperpolarizing shift of the RMP. Given the inter-subject variability of the excitability measures and *in vivo* nature of the recording, the simulated potential in each group might not be accurate. However, the hyperpolarizing shift of the resting potential by HLU could have resulted in axonal hypoexcitability. Suppression of persistent Na^+^ current might have decreased the transmembrane ionic flow, failing to maintain the proper excitability (Lin and Baines, 2015).

### What suppresses Na^+^ current during hindlimb unloading?

Na^+^ channels play an integral part in determining membrane excitability and generation and transmission of action potentials. Therefore, their impairment is associated with a number of neurological conditions, such as tetrodotoxin intoxication by pufferfish (Kiernan et al., 2005) and critical illness neuropathy by inactivation of Na^+^ channels (Novak et al., 2009).

Although the exact mechanism for downregulating INaP and the transient Na^+^ current by HLU is elusive, there are several hypotheses: (1) Alternative splicing of RNA and translational repression of Na^+^ channels are activity-dependent; thus INaP and overall membrane excitability are activity dependent (Lin and Baines, 2015); (2) Secretion of brain-derived neurotrophic factor (BDNF) is activity dependent (Karpova, 2014). Lack of anti-gravity activity alters signaling mechanisms such as transactivation of an intracellular domain of tropomyosin receptor kinase B (TrkB) and subsequently changes the open probability of Na^+^ channels (Wetzel et al., 2013).

### Alterations of other channels and pumps

In the myelinating axons, multiple ion channels/currents function as major determinant of RMP (Krishnan et al., 2009). Common characteristics of these are channel properties that are open at the subthreshold range to enable rather constant influx/efflux of ions (i.e., slow activating and deactivating kinetics). Besides persistent Na^+^ current as discussed earlier, “slow” K^+^ current and H current have been focused. “Slow” K^+^ current is mediated by KCNQ (Kv7) channels that are present in the node of Ranvier (Schwarz et al., 2006). Opening of HCN channels causes Ih (H current) that are activated by hyperpolarization (Howells et al., 2012). Besides setting RMP, HCN channels also play an important role in pace-making. Although theoretically important, these channels/currents were not significantly different between the controls and the HLU animals, thus further discussion will be omitted. Other than changes in the Na^+^ channels, the modeling study suggested the following possible alterations by HLU: (1) increased GBB, (2) increased leak conductance, and (3) decreased pump current, that are discussed below for their potential significance.

GBB is related to insulation or paranodal sealing of the myelin sheath. Its abnormality might be associated with pathological changes in the paranodal region. Indeed, animals after HLU were reported to show thinner myelin in the hindlimb, although there was no evidence to suggest abnormal nodal or paranodal morphology (Canu et al., 2009).

Ion channels that are open are responsible for maintaining a hyperpolarizing RMP, crucial in neural functions. Activation of leak (or background) K^+^ channels drives the membrane potential closer to the K^+^ equilibrium potential of ~−90 mV, and therefore reduces excitability (Honore, 2007). Two-pore K^+^ channels are responsible for the leak currents that are present in the spinal cord and dorsal root ganglia, but their existence in the peripheral motor axons has not been fully explored (Gonzalez et al., 2012). Although two-pore K^+^ channels are modulated by multiple factors (e.g., intracellular pH, stretch, temperature, protein kinases, and volatile anesthetic agents; Honore, 2007), the authors are not aware of any study regarding the effect of muscle disuse or microgravity on the expression of these channels.

Na^+^-K^+^-ATPase is a crucial protein responsible for the electrochemical gradient across the cell membrane. Its impairment leads to membrane depolarization and entry of Ca^2+^ that triggers neurotransmitter release and neuronal death (de Lores Arnaiz and Ordieres, 2014). Suppression of IPumpNI reduces late subexcitability, consistent with the observation with HLU.

Overall, any of the possibilities (increased leak current, increased GBB, and decreased IPumpNI) significantly affect waveforms in TE that was not observed in the present study. As the simulation showed (Table 2), at least two of these parameters are considered to be affected, resulting in the net effect of unchanged TE waveform.

### Limitations

This study has some limitations. First, the etiology of muscle atrophy as evident from decreased amplitudes of CMAPs by HLU could be multifactorial. Primary muscle atrophy by HLU has been reported to be caused by multiple factors (e.g., disruption in the balance between protein synthesis and breakdown; Narici and de Boer, 2011). It is unclear whether the observed axonal excitability and channel dysfunctions are directly relevant to clinical symptoms and signs. Second, because this is an *in vivo* physiological study of the motor axons, there is no confirmation by pathological techniques or cellular physiology. Furthermore, similar to the previous study (Boërio et al., 2009), the excitability waveforms in the present protocol (recorded from the foot muscle and stimulating the sciatic nerve) do not reproduce those recorded in human subjects, characterized by smaller threshold change by long hyperpolarization and upward shift of RC, possibly due to relative membrane depolarization of the stimulated distal sciatic nerve. Third, given the short duration of HLU and reversible suppression of persistent Na^+^ current after release, it could be elusive whether the suppressed sodium current leads to axonal degeneration. Fourth, as mentioned in the preceding paragraph, confirmatory data are lacking regarding the potential abnormality associated to current leaking. GBB is related to insulation or paranodal sealing of the myelin sheath. Immunostaining of the peripheral axons showed normally located Na^+^ and K^+^ channels at the node and juxtaparanodal regions, respectively, as well as unchanged nodal and paranodal lengths, suggesting grossly intact paranodal sealing (Canu et al., 2009). However, detailed information by electron microscope has been lacking. The responsible channels for the leak current has yet to be determined in the peripheral axons. The activity of axonal Na^+^-K^+^-ATP is not straightforward, because measurement of a biological activity or quantification of mRNA of a dissected peripheral nerve may be contaminated by the presence of non-axonal tissues. The effect of a modulating drug on hindlimb uploaded animals might clarify the potential pathogenesis of these factors. Additionally, we did not quantify mRNA for all the alpha- and beta- subunits of the Na^+^ channels, because of the small amounts of the extracted sciatic nerve tissues. More extensive genetic quantification should be available by a future study using a rat or other larger animals.

### Clinical implication

From a diagnostic standpoint, the threshold-tracking technique is a non-invasive electrophysiological method that can be applied to patients at their bedside and can be performed within 20 min. Because disuse muscular atrophy has poor prognosis, it could function as a biomarker to predict high-risk individuals early during hospitalization to prevent irreversible muscular atrophy. Obviously further clinical studies are needed to elucidate such possibilities.

## Author contributions

CB performed all *in vivo* recordings and analyzed the data. HN designed experiments, drafted the manuscript, and oversaw the project. TK and CB performed the qRT-PCR experiment. SH and RO performed all *in vivo* experiments. YS, AM, and YO interpreted the data. RK supervised this study.

### Conflict of interest statement

The authors declare that the research was conducted in the absence of any commercial or financial relationships that could be construed as a potential conflict of interest.

## Supplementary material

Supplemental Figure 1 **Representative waveforms of the axonal excitability tests and definitions of the parameters: threshold electrotonus (A), recovery cycle (B), and current-threshold relationship (I/V) (C)**. Data are modified from a paper by Nodera and Rutkove.(Nodera et al., 2011).

Supplemental Figure 2 **Interval changes of the strength-duration time constant (SDTC) after releasing from HLU**. The lower SDTC by HLU than the control animals became similar 3 weeks after releasing from HLU (*P* < 0.05, Wilcoxon signed rank test).

Supplemental Figure 3 **Waveforms of the modeling study. First, the conductance and currents were optimized to fit the excitability waveforms of the control animals (dashed lines)**. Second, the parameters were optimized to reduce the error to the obtained waveforms in the unloading group (open circles). The simulated waveforms (solid lines) faithfully reproduce the difference in the two groups [i.e., smaller late subexcitability (arrowhead in **C**) and smaller strength-duration time constant (arrow in **D**) in the unloading group than the controls].

Supplemental Figure 4 **Agarose gel electrophoresis of the qRT-PCR products revealed that the cDNA fragment of 150-bp (upper panel A), 111-bp (upper panel B) and 144-bp (upper panel C), corresponding to the predicted mouse Nav1.1, Nav1.2 and ribosomal protein S16 transcript, respectively**. Melting profile demonstrates symmetric melting curves with single sharp peak, indicating that the PCR assay is optimized (lower panels **A–C**).

Supplemental Table 1 **Information of PCR primers used in the study**.

Supplemental Table 2 **List of the parameters for ion conductance and currents to best fit the recordings of the control and hindlimb unloading groups**.

## Abbreviations

ALS: Amyotrophic lateral sclerosis
BDNF: brain-derived neurotrophic factor
CMAP: compound muscle action potential
GBB: Barrett-Barrett conductance
HCN channel: hyperpolarization-activated cyclic nucleotide-gated channel
HLU: hindlimb unloading
Ih: hyperpolarization-activated current
INap: persistent Na^+^ current
I/V: current-threshold relationship
PCR: polymerase chain reaction
qRT–PCR: quantitative reverse transcription–polymerase chain reaction
RC: recovery cycle
RMP: resting membrane potential
RT: reverse transcription
SDTC: strength-duration time constant
TE: threshold electrotonus
TrkB: tropomyosin receptor kinase B
VGSC: Voltage-gated Na^+^ channels.
