# Supplementary figures and images for: Impaired Axonal Na+ Current by Hindlimb Unloading: Implication for Disuse Neuromuscular Atrophy
Source: Front Physiol. 2016 Feb 16;7:36. doi: 10.3389/fphys.2016.00036 (PMC4754663; doi:10.3389/fphys.2016.00036)

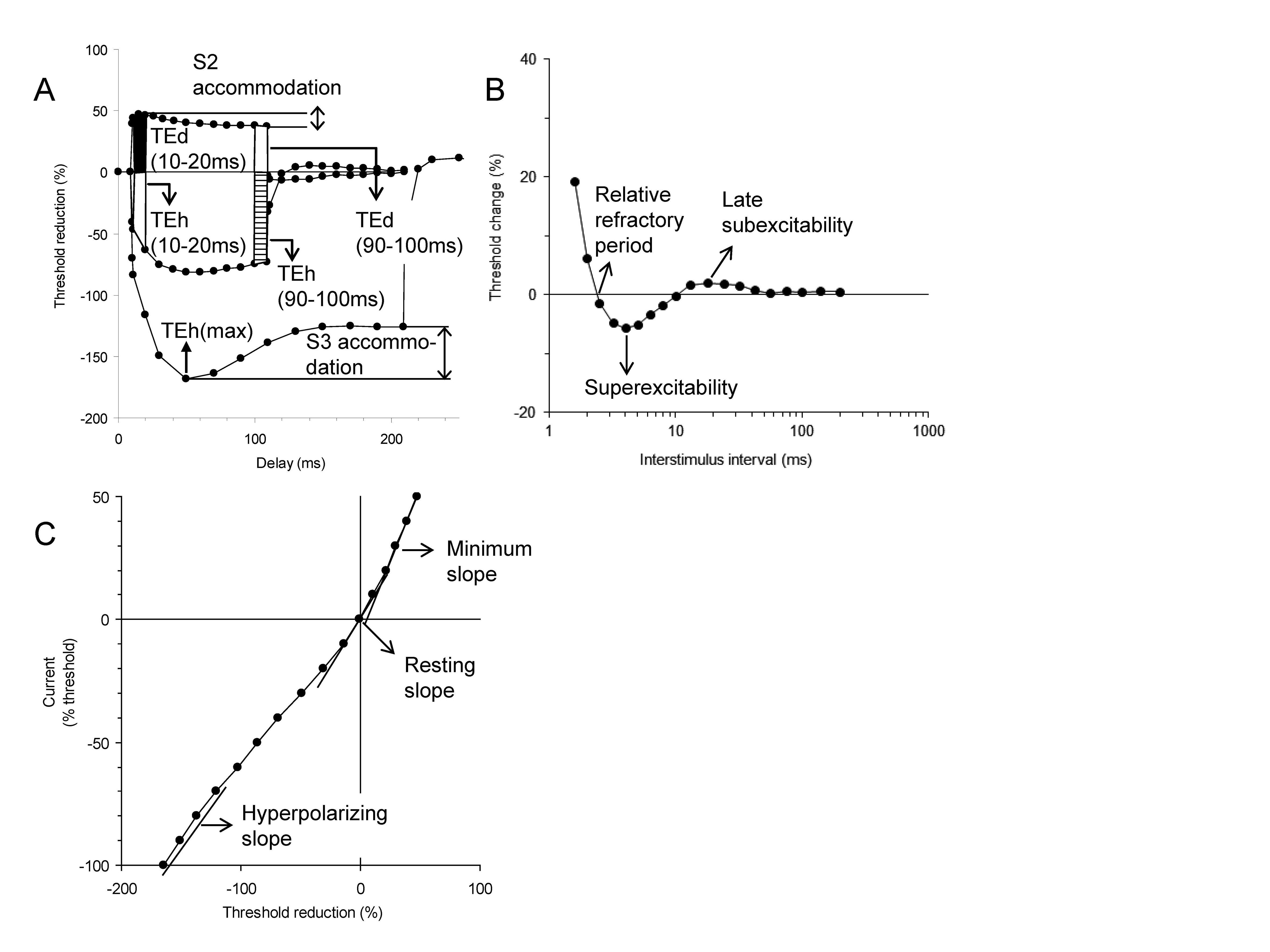

Supplement: Supplemental Figure 1 — Representative waveforms of the axonal excitability tests and definitions of the parameters: threshold electrotonus (A), recovery cycle (B), and current-threshold relationship (I/V) (C). Data are modified from a paper by Nodera and Rutkove.(Nodera et al., 2011). [file Image1.TIFF]

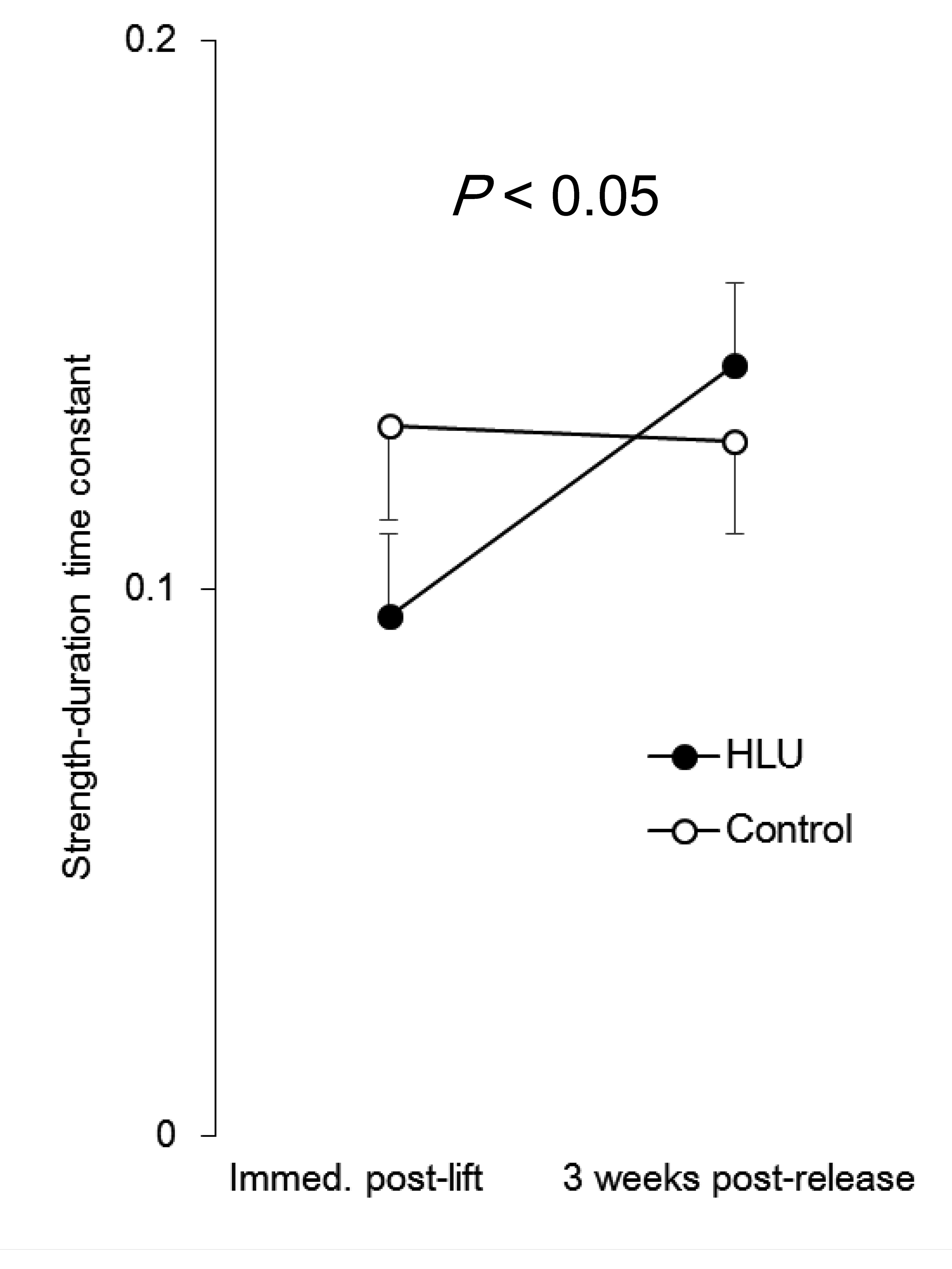

Supplement: Supplemental Figure 2 — Interval changes of the strength-duration time constant (SDTC) after releasing from HLU. The lower SDTC by HLU than the control animals became similar 3 weeks after releasing from HLU (P < 0.05, Wilcoxon signed rank test). [file Image2.TIFF]

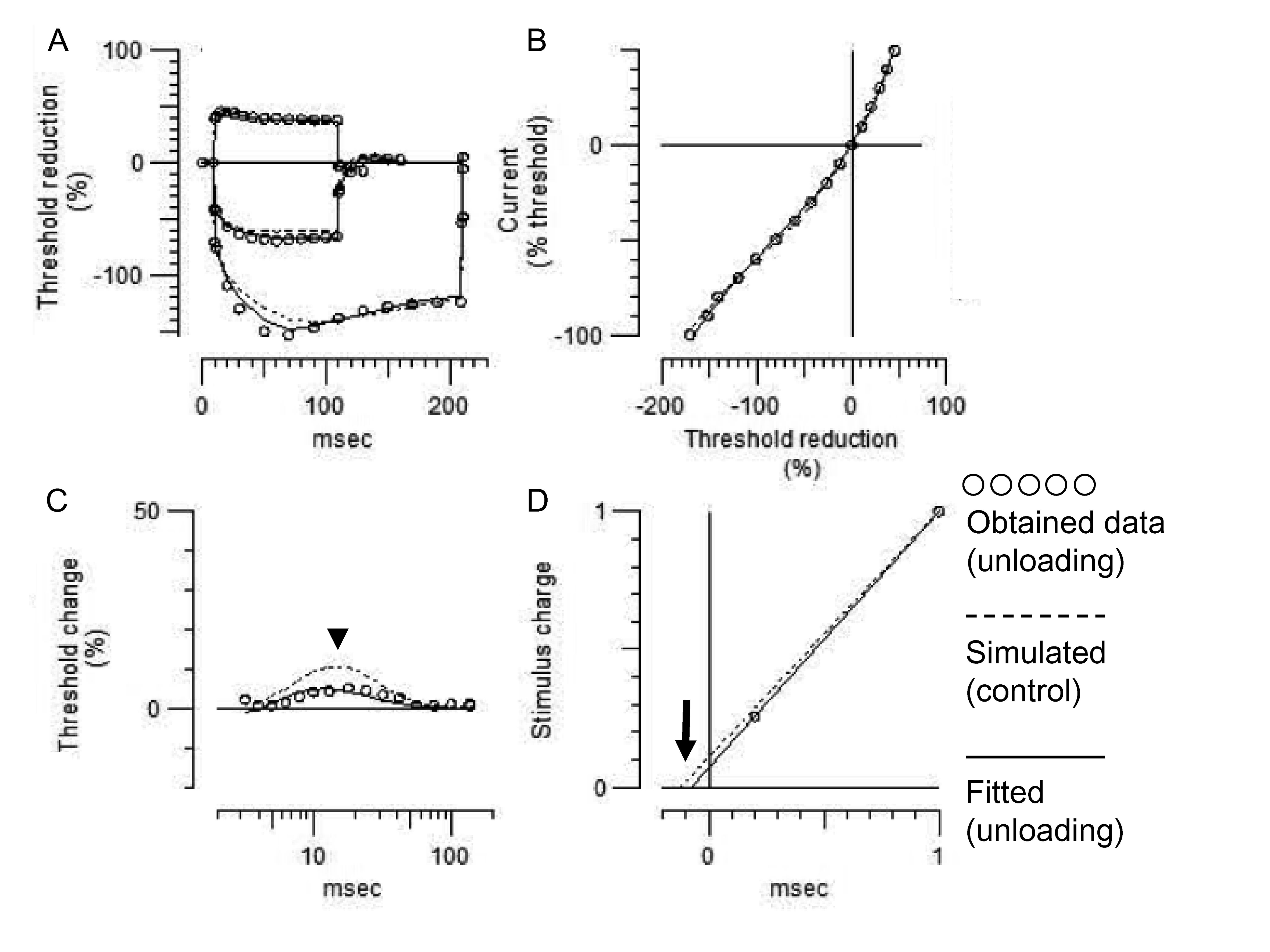

Supplement: Supplemental Figure 3 — Waveforms of the modeling study. First, the conductance and currents were optimized to fit the excitability waveforms of the control animals (dashed lines). Second, the parameters were optimized to reduce the error to the obtained waveforms in the unloading group (open circles). The simulated waveforms (solid lines) faithfully reproduce the difference in the two groups [i.e., smaller late subexcitability (arrowhead in C) and smaller strength-duration time constant (arrow in D) in the unloading group than the controls]. [file Image3.TIFF]

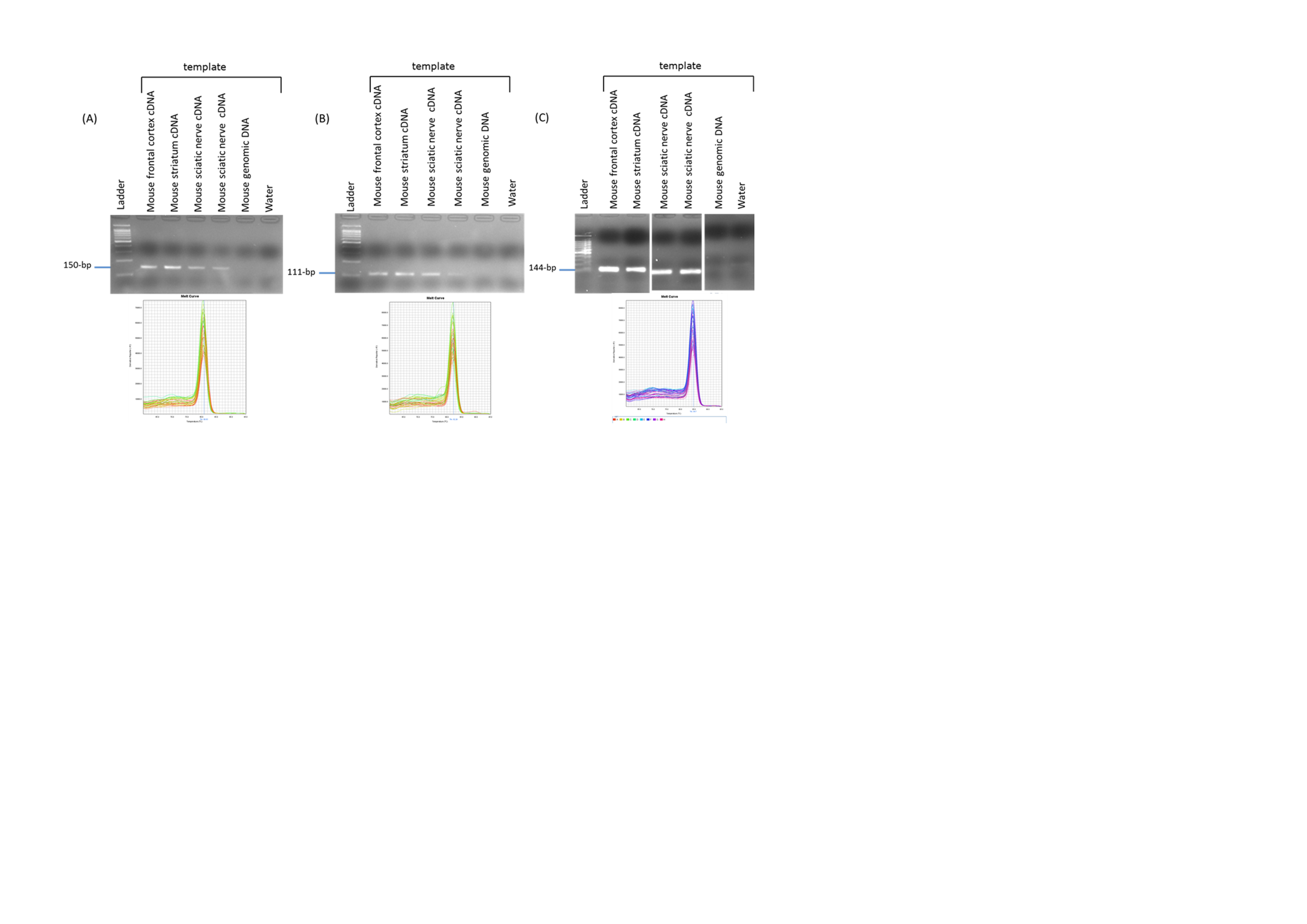

Supplement: Supplemental Figure 4 — Agarose gel electrophoresis of the qRT-PCR products revealed that the cDNA fragment of 150-bp (upper panel A), 111-bp (upper panel B) and 144-bp (upper panel C), corresponding to the predicted mouse Nav1.1, Nav1.2 and ribosomal protein S16 transcript, respectively. Melting profile demonstrates symmetric melting curves with single sharp peak, indicating that the PCR assay is optimized (lower panels A–C). [file Image4.TIFF]
