# Supplementary material for: Impaired Axonal Na+ Current by Hindlimb Unloading: Implication for Disuse Neuromuscular Atrophy
Source: Front Physiol. 2016 Feb 16;7:36. doi: 10.3389/fphys.2016.00036 (PMC4754663; doi:10.3389/fphys.2016.00036)
Supplement: Supplemental Table 1 — Information of PCR primers used in the study. [file Table1.docx]

Supplemental Table 1: Information of PCR primers used in the study

|  |  |  |  | **Product length (bp)** |  |
| --- | --- | --- | --- | --- | --- |
|  | **Gene** |  | **Primer sequence (5’-3’)** | **Amplicon size (base pair)** | **Accession No.** |
| mNav1.1-F | Nav1.1 | Forward | AACAAGCTTCATTCACATACAATAAG | 150 | NM_018733 |
| mNav1.1-R |  | Reverse | AGGAGGGCGGACAAGCTG |  |  |
| mNav1.2-F | Nav1.2 | Forward | GGGAACGCCCATCAAAGAAG | 111 | NM_001099298 |
| mNav1.2-R |  | Reverse | ACGCTATCGTAGGAAGGTGG |  |  |
| mRiboS16-F | Housekeeping gene | Forward | CTGGAGCCTGTTTTGCTTCTG | 144 | NM_013647 |
| mRiboS16-R | Mouse ribosomal protein S16 | Reverse | TGAGATGGACTGTCGGATGG |  |  |
