# Supplementary material for: Impaired Axonal Na+ Current by Hindlimb Unloading: Implication for Disuse Neuromuscular Atrophy
Source: Front Physiol. 2016 Feb 16;7:36. doi: 10.3389/fphys.2016.00036 (PMC4754663; doi:10.3389/fphys.2016.00036)
Supplement: Supplemental Table 2 — List of the parameters for ion conductance and currents to best fit the recordings of the control and hindlimb unloading groups. [file Table2.docx]

Supplemental Table 2: List of the parameters for ion conductance and currents to best fit the recordings of the control and hindlimb unloading groups

| Parameter | Description | Unloading | Control |
| --- | --- | --- | --- |
| EIR (mV) | Internodal resting membrane potential | -93.2 | -88.5 |
| ENR (mV) | Nodal resting membrane potential | -93.2 | -89.6 |
| PNaN (cm^3^s^-1^ × 10^-9^) | Nodal Na^+^ permeability | 4.0 | 6.85 |
| PNaP (%) | Percent persistent Na^+^ | 0.15 | 0.30 |
| GKsN (nS) | Max. nodal conductance of slow K^+^ channels | 44.5 | 49.4 |
| GKsI (nS) | Max. internodal conductance of slow K^+^ channels | 665 | 272 |
| GKfN (nS) | Max. nodal conductance of fast K^+^ channels | 6.5 | 6 |
| GKfI (nS) | Max. internodal conductance of fast K^+^ channels | 909 | 717 |
| GH (nS) | Internodal H conductance | 33 | 33 |
| GLk (nS) | Nodal and internodal leak conductance | 18.9 | 7.3 |
| GBB (nS) | Barrett-Barrett conductance | 60.3 | 46.5 |
| IPumpNI (nA) | Nodal and internodal pump current | 0.023 | -0.049 |
